# Acoustical and Optical Determination of Mechanical Properties of Inorganically-Bound Foundry Core Materials

**DOI:** 10.3390/ma13112531

**Published:** 2020-06-03

**Authors:** Philipp Lechner, Georg Fuchs, Christoph Hartmann, Florian Steinlehner, Florian Ettemeyer, Wolfram Volk

**Affiliations:** 1 Chair of Metal Forming and Casting, Technical University of Munich, Walther-Meissner-Strasse 4, 85748 Garching, Germany; georg.fuchs@utg.de (G.F.); christoph.hartmann@utg.de (C.H.); florian.steinlehner@utg.de (F.S.); wolfram.volk@utg.de (W.V.); 2 Fraunhofer Research Institute for Casting, Composite and Processing Technology IGCV, Walther-Meissner-Strasse 4, 85748 Garching, Germany; Florian.ettemeyer@igcv.fraunhofer.de

**Keywords:** inorganic sand core materials, elastic properties, fracture strength, fracture strain, water-glass, 3-point-bending, Young’s modulus, shear modulus, Poisson’s number

## Abstract

Inorganically-bound sand cores are used in many light-metal foundries to form cavities in the cast part, which cannot be realised by the mould itself. To enable FEM simulations with core materials, their mechanical properties have to be measured. In this article, we adapt methods to determine the Young’s and shear modulus, the Poisson ratio and the fracture strain of sand cores. This allows us to fully parametrise an ideal brittle FEM model. We found that the Young’s and shear modulus can be obtained acoustically via the impulse excitation technique. The fracture strain was measured with a high-speed camera and a digital image correlation algorithm.

## 1. Introduction

A rising number of light-metal foundries are using inorganically-bound core materials instead of organically-bound ones, due to stricter environmental laws and the need for eco-friendly production processes [1]. A typical industrial application for inorganic cores is the casting of motor components in the automotive industry [2]. The cores consist of sand and a binder agent, which holds the individual particles together and forms a porous material, which can be used for foundry moulds and cores [3]. Inorganic sodium silicate binders (waterglass) do not combust during the casting, while organic binders emit a number of hazardous combustion products. However, the absence of a combustion process also means that more binder remains in the cast-in sand core. After a cooling period, the sand cores have to be removed via high-energy hammer impacts to the cast component, which is called de-coring [4].

With inorganic binders, this process is more energy-intensive and straining for the cast components, due to the higher amount of remaining binder. In order to minimise the necessary effort to remove the core from the cast component, one needs to find out which de-coring process parameters are energy- and strain- minimal. This directly leads to the goal of generating de-coring simulations which allow to evaluate if the cast part is de-corable. Subsequently, such simulations can be utilised to optimise the process and thus reduce the strain on the component. The first step of building such a mechanical simulation is to determine the basic material parameters, in order to build a material model. Lechner et al. showed that inorganically-bound core materials behave like a brittle material [5]. For a brittle material, the basic material parameters for a simulation are Young’s modulus, shear modulus, fracture stress and fracture strain. There are numerous articles which study mechanical properties of inorganically-bound core materials. Most focus on the mechanical strength and the influence of environment and heat treatment on it [6]. However, research also exists, which studies the Young’s modulus of core materials. Stauder et al. determined fracture stress and Young’s modulus with 3-point-bending (3PB) experiments [7]. Griebel et al. showed that strain should be measured optically, when determining Young’s Modulus with a 4-point-bending (4PB) experiment [8]. They further showed that the Young’s modulus is dependent on the applied load, and decreases towards the point of fracture.

Schneider et al. simulated the Young’s modulus by modelling the microstructure of the sand-binder-compound. Furthermore, they validated their simulation with an ultrasound through-transmission test [9]. However, to our knowledge, there is no existing literature on the shear modulus or Poisson ratio of inorganically-bound core materials. In this article, we propose methods to efficiently determine a complete and consistent parameter set for a brittle material model for inorganic core materials.

## 2. Materials and Methods

### 2.1. Specimens

The specimens used for this work were produced on a Loramendi SLC2 25L core-shooting machine (Loramendi S.Coop., Vitoria-Gasteiz, Spain) with a heated core box and a hot-gas drying device. A H 32 quartz sand (Quarzwerke GmbH, Frechen, Germany) was bound with an inorganic Inotec binder system (ASK Chemicals GmbH, Hilden Germany). The binder system consists of a liquid component EP 4158 (2 wt%) and a powder additive TC 4500 (1.6 wt%), which are measured relative to the sand mass. The temperatures were set to 155 °C core box and 220 °C gas temperature. The specimens were stored for 16 h at 20 °C and 10% humidity after production. The dimensions are 173.5×22.8×22.8 mm.

### 2.2. Determination of Elastic Properties with the Impulse Excitation Technique

The impulse excitation technique allows the identification of natural frequencies of the tested specimen [10]. An impact, in our case induced by a hammer, excites the specimen with a short impulse, and causes the subsequent approximately free oscillation of the specimen measured [11]. There are various possibilities for measuring this oscillation. The sound can be recorded with a microphone or the specimen can be tracked with a contact-less laser vibrometer or a piezo crystal [12]. Furthermore, the induced strains can be measured using strain gauges. Regardless of the method, the data are recorded in the time domain and Fourier-transformed to the frequency domain. In the subsequent spectrum, every excited natural frequency is represented by a peak.

With the dimensions of the cuboid specimen and its density, the obtained natural frequency peaks can be used to calculate elastic properties of the material used. There are several analytic solutions for the Young’s modulus and the shear modulus. We use the following equations to calculate the Young’s modulus *E*, the shear modulus *G* and the Poisson ratio ν in this article [13]:

(1) $$E=0.946\left(\frac{mf_{f_{1}}^{2}l^{3}}{bh^{3}}\right)\left(1+6.585{\left(\frac{h}{l}\right)}^{2}\right)$$

(2) $$G=\left(\frac{4lmf_{t_{1}}^{2}}{bh}\right)\left(\frac{\left(h/b)+(b/h\right)}{4\left(h/b\right)-2.52{\left(h/b\right)}^{2}+0.21{\left(h/b\right)}^{6}}\right)$$

(3) $$\nu =\frac{E}{2G}-1$$

where *m* is the mass of the specimen, *l* the length, *b* the width and *h* the height. $f_{f_{1}}$, $f_{l_{1}}$ and $f_{t_{1}}$ are the first bending, longitudinal and torsional eigenfrequencies, respectively.

### 2.3. Test Setup for Acoustic Determination of Elastic Properties

The three main components of the acoustic test bench are the automatic hammer, the control unit which controls the microphone (S241 from Superlux, New Taipei, Taiwan) and the hammer, and the support structure with two parallel steel cords. The automatic hammer is actuated by a stepper motor and has a steel tip. One challenge with an automatic hammer is to avoid multiple hits of the specimen. Our hammer is made of ABS polymer, which means that it is flexible. The stepper motor starts retracting the hammer before it hits the specimen. This leads to a deflection of the hammer and a subsequent impact on the specimen, while the stepper motor is already moving backwards. This allows to retract in good time after the first hit, thus avoiding multiple hits of the specimen. The specimen rests on two parallel steel wires. The control unit consists of a Raspberry Pi, which controls the microphone and the stepper motor via custom python software. The sampling frequency of the system is 96 kHz which leads to a resolution in the frequency domain of 6 Hz.

**Figure 1:**
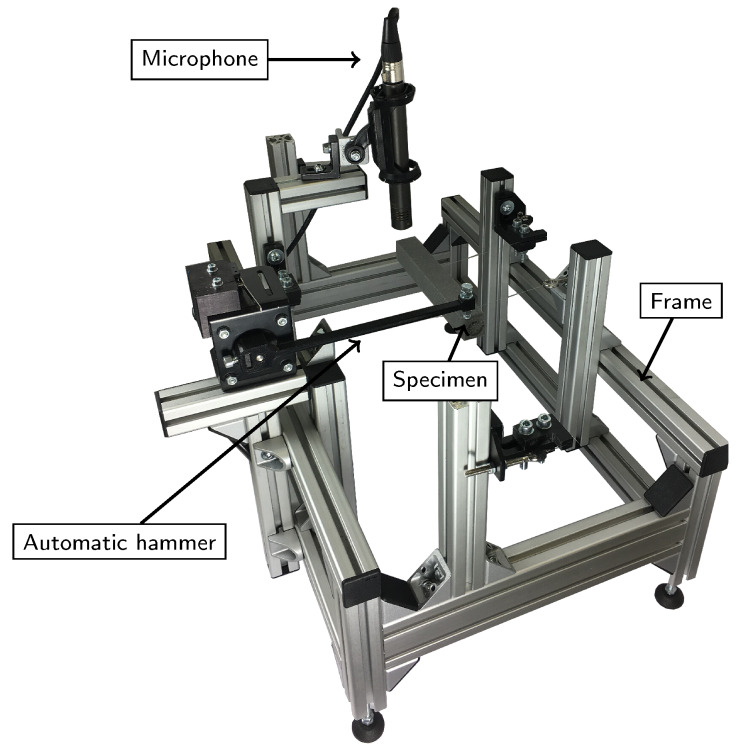
Test-bench for determining elastic properties via impulse excitation technique. The main components are a microphone, a specimen, an automatic hammer system and the frame of the test-bench.

**Figure 2:**
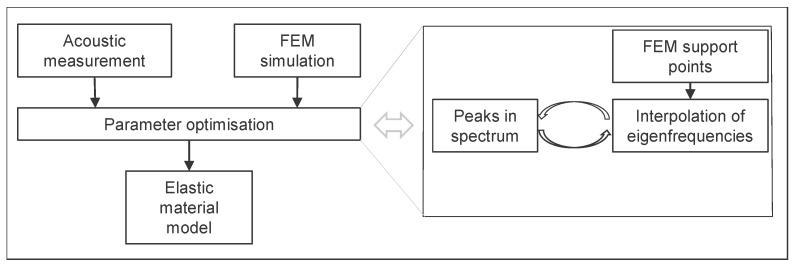
Algorithm for determining elastic parameters via impulse excitation technique and FEM simulation. The measured and calculated eigenfrequencies are compared and the Young’s modulus and Poisson ratio are optimised such, that the error between the measured and the calculated eigenfrequencies is minimal.

Figure 1 shows the test bench. The impact position was chosen in one corner of the beam in order to excite both flexural and torsional eigenmodes. Figure 2 shows the algorithm used in this article to determine elastic properties via impulse excitation technique and FEM simulation. The measured and calculated eigenfrequencies are compared and the Young’s modulus and Poisson ratio are optimised such that the error between the measured and the calculated eigenfrequencies is minimal. In order to achieve a mechanical representation of the real elastic behaviour as accurate as possible, more than the first flexural and torsional frequency can be used to calculate the Young’s modulus and the Poisson ratio. We simulated natural frequencies at support points for the following tupel of Young’s modulus and Poisson ratio [(5500,0);(5500,0.28);(6000,0.14);(6500,0);(6500,0.28)] in FEM. The results were used to fit a linear model, which calculates natural frequencies in the two-dimensional space of the support points. Additionally, we introduced an error function which calculates the root mean square error (rms) for multiple peaks between experiment and simulation. This model can be optimised such that the rms is minimal and thus fits the elastic model optimally to the obtained experimental data. This was performed via linear optimisation in Matlab (MathWorks Inc., Natick, MA, USA).

### 2.4. FEM Calculation of Natural Frequencies

**Figure 3:**
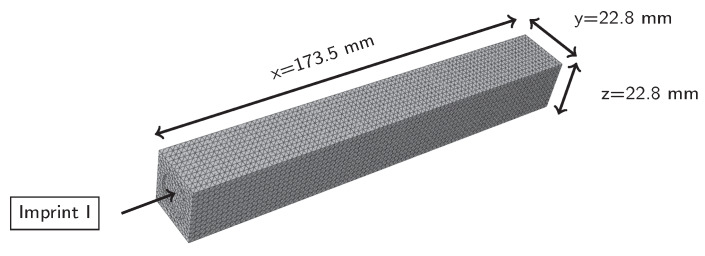
Meshed specimen for FEM calculation of natural frequencies and eigenmodes.

**Figure 4:**
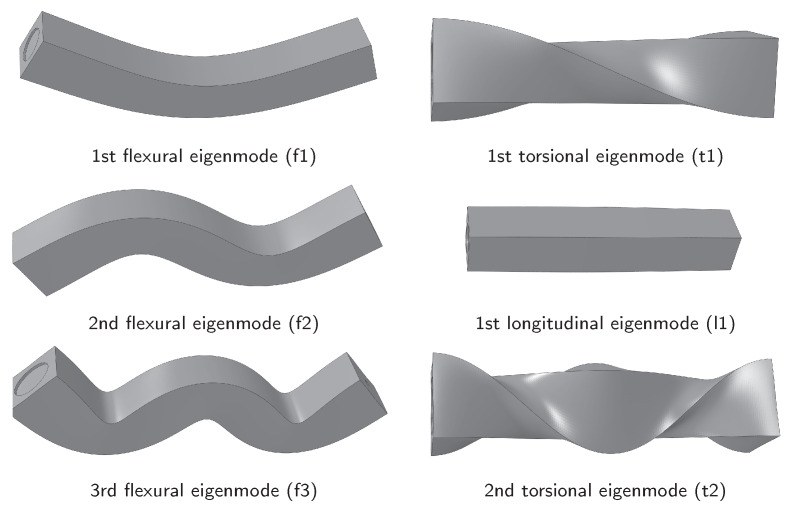
Eigenmodes 1–6 of the specimen obtained by FEM simulation.

The natural frequencies and eigenmodes of the specimen were calculated with the FEM-Software Abaqus 2018 (Dassault Systems, Velizy-Villacoublay, France). The model is shown in Figure 3. The specimens were measured and modelled, including minor production inaccuracies: for example the imprint I on the front side of the beam is due to the shooting nozzle. The model was meshed with tetrahedron elements with an approximate global size of 2 mm. The calculation was done with a Lanczos solver. Figure 4 depicts FEM results for eigenmodes 1–6. The simulation shows that each flexural eigenmode is split in two very close neighbours, due to the asymmetry on the front side of the beam, which results from the production process. For reasons of simplicity, only the first one of the neighbour-pairs will be considered in this article.

### 2.5. Digital Image Correlation Algorithm and High-Speed Imaging

For simplicity, reliability and flexibility reasons, digital image correlation (DIC) has been established as the standard for optical deformation analysis in experimental mechanics of solids. Digital image correlation rests on registration algorithms based on correlation functions. Based on the merging, relative displacements between subsequent images are calculated. The method has been extensively investigated and various task-specific correlation criteria are proposed and used in different fields [14]. Integer-pixel accuracy can be achieved using simple correlation criteria. For sub-pixel resolution data, interpolation is necessary, for example coarse-to-fine searching [15], peak-finding algorithms [16], genetic algorithms [17], gradient-based algorithms [14], B-splines, artificial neural networks [18] and finite elements [19]. Given adequate image acquisition, DIC features scale-invariance leading to a large field of applications.

**Figure 5:**
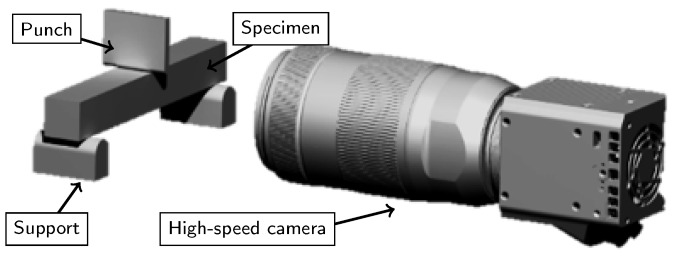
Test setup for a 3-point-bending experiment with high-speed imaging. The camera is oriented orthogonal to the specimen’s surface.

For the DIC analysis in this article, the ICGN algorithm presented by Pan et al. has been adapted [20]. No artificial speckle pattern has to be applied, since the natural texture of the specimen suffices for adequate analysis. First-order shape functions for warping, a subset size of 15×15 pixels and a discrete cosine transform filter proposed by Garcia are used [21]. Further details on the precision, accuracy and present implementation of the algorithm are presented by Hartmann et al. [22]. A high-speed camera (Os3-S2 from IDT, Pasadena, CA, USA) with magnification optics (Ultrasonic from Canon, Tokyo, Japan) is used for the acquisition of the deformation (8-bit grey-scale images). The images are recorded at a frequency of 8500 Hz. A macro cold light source (Xenon Nova 300 W, Storz, Tuttlingen, Germany) is used for illumination. The test setup is shown in Figure 5.

### 2.6. Test Setup for Measuring Fracture Stress and Strain

We used 3-point-bending (3PB) experiments to determine the tensile fracture stress and strain. The distance between the supports was 150 mm. The experiments were carried out on a Zwick Z20 (ZwickRoell GmbH & Co. KG, Ulm, Germany) equipped with a 20 kN force sensor. The stress was calculated from the maximum force at fracture, via beam theory according to [23]:

(4) $$\sigma_{3PB}=\frac{3F_{3PB}l}{2bh^{2}}$$

where $\sigma_{3PB}$ is the maximum stress in the beam. $F_{3PB}$ is the applied force and *l* is the distance between the supports. *b* is the width and *h* the height of the beam.

The fracture strain was determined using 3PB as well. The high-speed camera was aligned to the area around the punch, which has the highest tensile stresses. The high-speed data was evaluated with the algorithm described in Section 2.5.

## 3. Experimental Results

### 3.1. Acoustical Determination of Elastic Parameters

**Figure 6:**
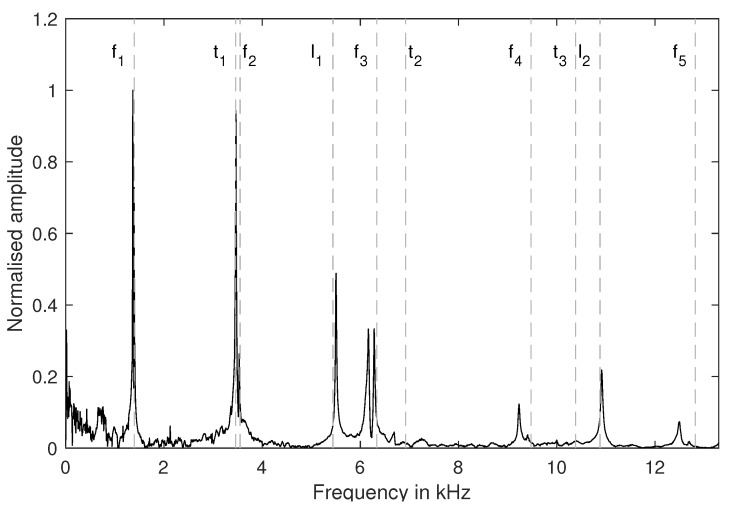
Spectral analysis of the impulse excitation data. The doted lines mark natural frequencies based on FEM calculations, with Young’s modulus and the shear modulus calculated analytically using f1 and t1, respectively.

We simulated the exact geometry of the beam to obtain the natural frequencies (<20 kHz) based on a purely elastic model, with Young’s modulus and the Poisson ratio as parameters. Comparing the simulation results with the measured spectrum allows the identification and matching of the specific eigenmodes with the peaks of the spectrum. Figure 6 shows a spectral analysis of one exemplary data set. We calculated the Young’s and shear moduli via Equations (1) and (2). Subsequently, the natural frequencies were calculated using FEM and the obtained elastic parameters. These natural frequencies are marked in the figure with dotted vertical lines and assigned to the specific eigenmodes with the sequence of eigenfrequencies from FEM. Until 5.5 kHz, the FEM results fit the measured peaks, while for higher frequencies they do not, especially for torsional frequencies.

The reason for this is that the mechanical reality differs from the material model utilised in the FEM-calculation. We studied the distance between successive natural frequencies to show this and calculated ratios of successive natural frequencies. This ratio is independent of the elastic parameters in simulation. For example, the first and second flexural frequency can both be used to determine the Young’s modulus. Their quotient is determined by the geometrical dimensions of the specimen. Since the dimensions of the measured beam and the simulation are approximately identical, this can serve as a criterion to show how well the elastic two-parameter model corresponds with the mechanical reality. Table 1 shows a comparison between the simulation and the experiment. There are small deviations between the simulation and the experiment, which is to be expected. However, one outlier can be identified. The ratio $f_{t1}/f_{t2}$ is significantly smaller in the experiment (1.81 compared to 2.00). To exclude the test setup as a reason for this behaviour, we changed the support to a cross, and included steel specimens as well. A variation of the supports had no influence on this effect, while a measurement using steel showed a ratio close to 2.00. This leads to the conclusion that this effect is in fact a typical behaviour for the inorganically-bound core materials used and that, contrary to the initial impression, the physics in the simulation corresponds better to the experiment at higher torsional frequencies.

**Figure 7:**
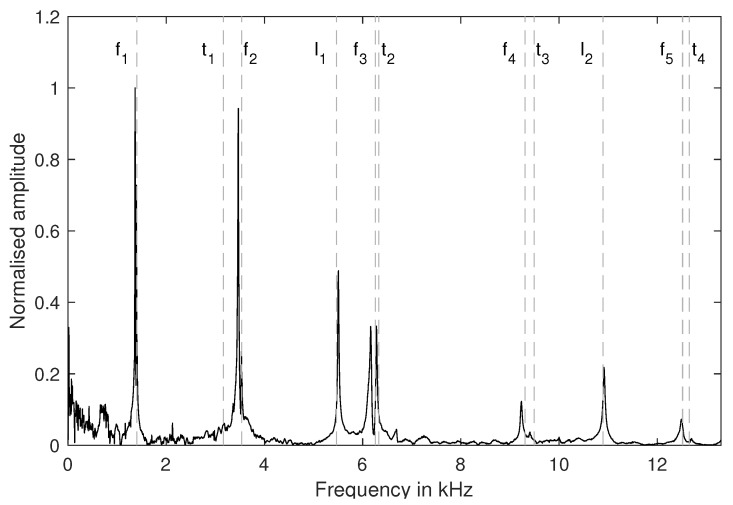
Spectral analysis of the impulse excitation data. The doted lines mark natural frequencies based on FEM calculations. The Young’s and shear modulus of the FEM material model result from a error minimisation between the peaks in the spectrum and the calculated eigenfrequencies (<12 kHz).

Figure 7 shows the results of the parameter optimisation, described in Section 2.4 (eigenmodes used: $f_{1}$, $f_{2}$, $f_{3}$, $t_{1}$, $t_{2}$, $t_{3}$, $l_{1}$, $l_{2}$). The simulated eigenmodes match significantly better to the measured spectrum, while $t_{1}$ remains an outlier. In Table 2 the elastic parameters for seven specimens S1–S7 are shown. The mean Young’s modulus is 5.75 GPa with standard deviation of 0.391 GPa. The mean shear modulus is 2.43 GPa with a standard deviation of 0.203 GPa. With Equation (3), this leads to a mean Poisson ratio of 0.183.

### 3.2. Optical Determination of Fracture Strain with High-Speed Images

**Figure 8:**
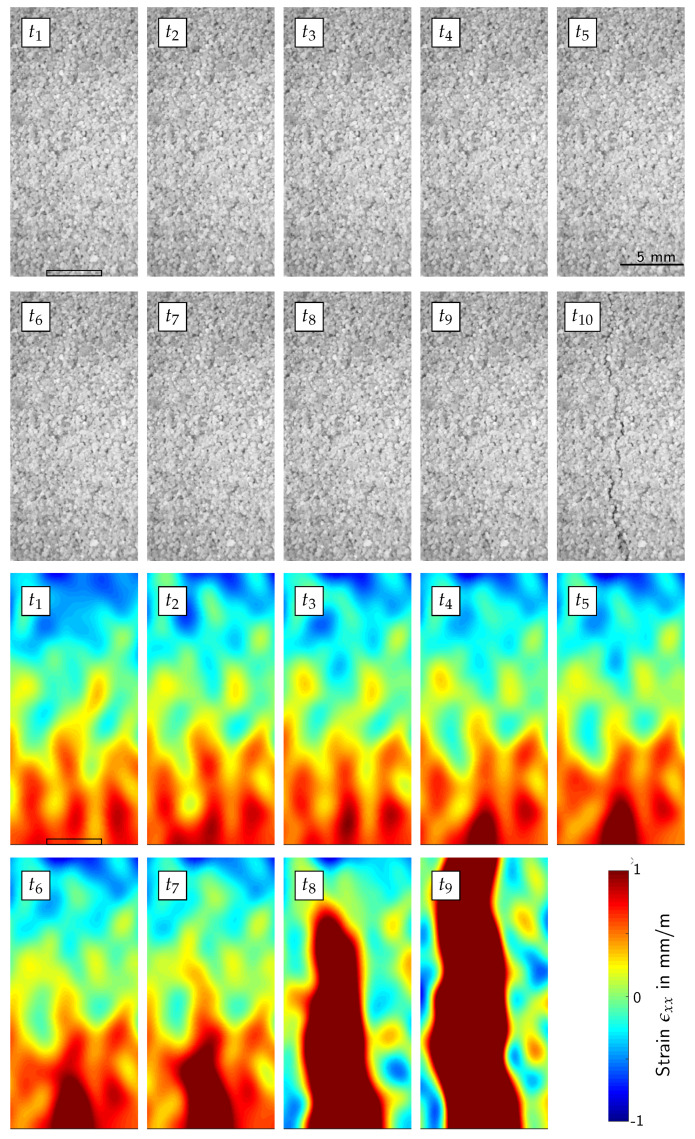
Strain calculation via digital image correlation for a 3-point-bending (3PB) experiment and 10 points in time ($t_{1}$–$t_{10}$). Only the area under the punch is analysed. Starting at $t_{2}$, the strain starts to localise until the fracture is complete at $t_{9}$, which is still undetectable by the human eye. We added a scale to the plots

In this section, we determine the fracture strain via digital image correlation and compare the result to a strain calculation based on the Young’s modulus and on the fracture force calculated using Equation (4) and Hooke’s law. Figure 8 shows the strain $\epsilon_{xx}$ in the beam in the area under the punch for nine different points in time, taken from one exemplary high-speed data set. It should be noted that positive stress translates to tension, and negative stress to compression.

As can be expected from a 3PB setup, there are tensile stresses in the lower half of the beam. Furthermore, the fracture is initiated under the punch at the lower beam surface, where analytically the highest tensile stresses are located. Starting from time $t_{2}$, the tensile stresses start to localise. In $t_{9}$, the fracture is complete. However, the fracture is not yet visible to the human eye. A fracture can be seen as soon as the beam starts to fall and the fracture opens up at time $t_{10}$. This image cannot be evaluated with the DIC-algorithm any more, since the fracture opening is too wide. For the fracture strain calculation, we used the last image without localising tensile strain. In our exemplary data set, this is $t_{1}$. The strain was calculated with a mean over a region of interest, which is placed at the location of the subsequent stress localisation, as marked with a rectangle in $t_{1}$. Please note, that only four specimens were recorded with the high-speed camera. The average of the evaluated results is 583 μm/m, and the results are shown in Table 2.

## 4. Discussion

Comparing the results of the eigenmode simulation and the acoustically obtained data shows that it is necessary to calculate the Young’s modulus and the shear modulus with multiple natural frequencies. This is in contrast to EN 843-2, which standardises the determination of elastic parameters of ceramics. There, the recommendation is to use the first flexural and the first torsional natural frequency to calculate the Young’s and shear moduli, respectively. Our results show that especially the first torsional eigenfrequency differs from the theoretical one obtained by mechanical calculations based on an elastic material model with two parameters (Young’s modulus and Poisson ratio). This effect is due to the complex material behaviour, which results from a inhomogeneous micro-structure of the sand-binder compound. However, taking more eigenmodes into account minimises this model error, since the simulation model fits quite well for higher frequencies and the flexural eigenmodes in general. Minimising the error between multiple peaks in the measured spectrum and the simulation leads to a stable evaluation of the acoustical data obtained with the impulse excitation technique.

In literature, only the Young’s modulus for inorganically-bound core materials can be found, while the shear modulus and Poisson ratio are yet to be determined. Furthermore, the Young’s modulus is highly influenced by the production parameters, environmental conditions, the type of sand and the binder used for the core shooting. This makes it difficult to validate the results obtained in the relevant literature. Griebel et al. determined the Young’s modulus of a different type of inorganic core to be approximately 7 GPa. They further showed that the Young’s modulus is influenced by the applied load, which leads to a Young’s modulus which is 20% lower upon fracture stress [8]. This is a known behaviour for various materials including metals, which has led to the practice of using only low-stress parts of the strain–stress curve for the calculation of the Young’s modulus [24]. Here, acoustical determination of the elastic parameters has two advantages over tensile tests. First, the impulse excitation technique only applies a low load on the specimen. Second, with a tensile test, the shear modulus cannot be determined without an additional torsion test.

Further validation of the acoustic results can be achieved by comparing them to the DIC data through Hook’s law.

(5) σ=Eϵ

where σ is the stress, *E* the Young’s modulus and ϵ the strain. Calculating the theoretical strain at fracture with the mean Young’s modulus and the mean fracture stress of our experiments yields a fracture strain of 468 μm/m, which is 20% lower than the actual measured fracture strain. Since the Young’s modulus is obtained with an acoustical low-load measurement, this is in close agreement with Griebel et al., and validates both, the strain and the Young’s modulus measurement [8].

Analysing the scatter of the results for fracture strain and fracture stress shows that the results of the fracture strain have significantly less scatter than the fracture stress. This is due to the calculation of the fracture stress, which assumes that the applied load is supported by a homogeneous specimen with spatially constant material behaviour. However, core-shooting leads to a complex micro-structure which does not always fulfil this assumption. Taking specimen S7 as example: The fracture stress is significantly lower, while the fracture strain is in close agreement with the other specimens. This leads to the assumption that the specimen had a local defect which was the origin of the premature fracture, and shows that the local measurement leads to a more precise and stable determination of the fracture behaviour.

However, the numerical requirements, the manual evaluation effort and the high data amount, which has to be stored and moved by the high-speed camera (10 GB per measurement) makes it a time-expensive method. For this reason, we propose to parametrise future FEM simulations with a fracture strain calculated from acoustical determined elastic parameters and a fracture stress, which is corrected by the factor 0.8 determined in this article.

## 5. Conclusions

In this article, we developed methods to determine all necessary physical properties for an ideal brittle material model of inorganically-bound core materials. The Young’s and shear moduli were determined via the impulse excitation technique. For the core material used in this article, the Young’s modulus was determined to be 5.75 GPa, while the shear modulus was 2.43 GPa. The fracture strain was determined using an universal testing machine and a high-speed camera to be 583 μm/m. With the test methods, proposed in this article, inorganically-bound core materials can be simulated with a fully parametrised ideal brittle model for the first time. This enables a simulation of complex stress states with FEM, and a comparison of the elastic simulation results with physical experiments. Our future work will include a study regarding the yield surface of inorganically-bound core materials, in order to characterise the plastic material behaviour in addition to the elastic model investigated in this article.

## Figures and Tables

**Table 1:** Ratios of successive natural frequencies obtained by simulation and experiment. The ratios are independent of the elastic parameters.

	$f_{f_{1}}/f_{f_{2}}$	$f_{f_{2}}/f_{f_{3}}$	$f_{f_{3}}/f_{f_{4}}$	$f_{t_{1}}/f_{t_{2}}$	$f_{t_{2}}/f_{t_{3}}$	$f_{l_{1}}/f_{l_{2}}$
Simulation	2.54	1.78	1.50	2.00	1.50	2.00
Experiment	2.59	1.75	1.50	1.81	1.50	1.98

**Table 2:** Results for elastic parameters of inorganically-bound core materials. The evaluation of the acoustical data was performed with an error minimisation for the Young’s modulus and the shear modulus.

Specimen No.	S1	S2	S3	S4	S5	S6	S7
Young’s modulus in GPa	6.18	5.92	5.62	5.15	5.89	-	5.89
Shear modulus in GPa	2.70	2.59	2.36	2.24	2.27	-	-
Strain in µm/m	597	-	-	578	-	580	576
Stress in MPa	2.92	2.95	2.84	2.78	2.51	2.70	2.21

## Abbreviations

The following abbreviations are used in this manuscript:

3PB	3-point-bending
4PB	4-point-bending
DIC	digital image correlation
FEM	finite element method
